# Cardioprotective effects of deferoxamine in acute and subacute cardiotoxicities of doxorubicin: a randomized clinical trial

**DOI:** 10.1186/s43044-023-00347-4

**Published:** 2023-03-24

**Authors:** Kosar Rahimi, Hamid Amoozgar, Soheila Zareifar, Mahdi Shahriari, Omid Reza Zekavat, Mehran Karimi, Gholamreza Fathpour, Fazl Saleh, Nader Shakibazad, Shayan Bordbar, Mohammadreza Bordbar

**Affiliations:** 1 Department of Pediatrics, Shiraz University of Medical Sciences, Shiraz, Iran; 2 Department of Pediatrics, and Divisions of Pediatric Cardiology, Shiraz University of Medical Sciences, Shiraz, Iran; 3 Hematology Research Center, Shiraz University of Medical Sciences, Shiraz, Iran; 4 Department of Pediatrics, and Divisions of Pediatric Hematology and Oncology, Shiraz University of Medical Sciences, Shiraz, Iran; 5 Students Research Committee, Shiraz University of Medical Sciences, Shiraz, Iran; 6 Bushehr University of Medical Sciences, Bushehr, Iran

**Keywords:** Cancer, Cardiotoxicity, Deferoxamine, Doxorubicin, Pediatric

## Abstract

**Background:**

Cardiotoxicity is a major concern following doxorubicin (DOX) use in the treatment of malignancies. We aimed to investigate whether deferoxamine (DFO) can prevent acute cardiotoxicity in children with cancer who were treated with DOX as part of their chemotherapy.

**Results:**

Sixty-two newly-diagnosed pediatric cancer patients aged 2–18 years with DOX as part of their treatment regimens were assigned to three groups: group 1 (no intervention, n = 21), group II (Deferoxamine (DFO) 10 times DOX dose, n = 20), and group III (DFO 50 mg/kg, n = 21). Patients in the intervention groups were pretreated with DFO 8-h intravenous infusion in each chemotherapy course during and after completion of DOX infusion. Conventional and tissue Doppler echocardiography, serum concentrations of human brain natriuretic peptide (BNP), and cardiac troponin I (cTnI) were checked after the last course of chemotherapy.

Sixty patients were analyzed. The level of cTnI was < 0.01 in all patients. Serum BNP was significantly lower in group 3 compared to control subjects (P = 0.036). No significant differences were observed in the parameters of Doppler echocardiography. Significant lower values of tissue Doppler late diastolic velocity at the lateral annulus of the tricuspid valve were noticed in group 3 in comparison with controls. By using Pearson analysis, tissue Doppler systolic velocity of the septum showed a marginally significant negative correlation with DOX dose (P = 0.05, r = − 0.308). No adverse effect was reported in the intervention groups.

**Conclusions:**

High-dose DFO (50 mg/kg) may serve as a promising cardioprotective agent at least at the molecular level in cancer patients treated with DOX. Further multicenter trials with longer follow-ups are needed to investigate its protective role in delayed DOX-induced cardiac damage.

*Trial registration* IRCT, IRCT2016080615666N5. Registered 6 September 2016, http://www.irct.ir/IRCT2016080615666N5.

**Supplementary Information:**

The online version contains supplementary material available at 10.1186/s43044-023-00347-4.

## Background

Anthracyclines are a group of chemotherapy agents, which are widely administered for pediatric hematologic malignancies and solid tumors; however, their associated adverse effects lead to decreased quality of life in survivors [1]. Early- or late-onset anthracycline-induced cardiotoxicity is a well-established concern, ultimately progressing to heart failure [2, 3]. The main mechanism of action of doxorubicin (DOX) as a potent anthracycline drug is interference with the DNA of neoplastic cells [4]. Various hypotheses have been proposed to explain the cardiotoxicity of DOX such as oxidative stress, altered molecular signaling, and promotion of apoptosis [5]. The main hypothesis supported by strong evidence is the production of reactive oxygen species (ROS) mediated via excess serum iron (SI) [6]. DOX-SI complexes are formed as these molecules chelate free iron within myocardial cells, which in turn contribute to the generation of highly reactive hydroxyl radicals [7]. Furthermore, DOX enhances the production of superoxide radicals. The consequent damage of ROS to the intracellular macromolecules disturbs vital pathways and leads to apoptosis or necrosis of cardiomyocytes [8].

Accordingly, iron-chelation therapy is applied to prevent the cardiotoxicity of anthracyclines in clinical settings [9]. Dexrazoxane is the only approved cardioprotective agent, which is validated in human studies and is administered in various cancers [10]. Prevention of ROS formation, binding to iron, and inhibition of DNA topoisomerase are proposed as the main mechanisms of action of dexrazoxane [11]. The potential efficacy of other iron chelators in the prevention of DOX-induced cardiotoxicity has been investigated in a few studies [12–14]. While dexrazoxane exerts its protective role via iron chelation, not all other iron-chelator agents have been proven to be effective. Deferoxamine (DFO) is another iron chelator with antioxidant properties. It binds with Fe, which leads to the prevention of redox cycling free iron and thus inhibits the potentiation of ROS [15]. DFO is a safe drug that is used in the treatment of iron overload disorders. As a result, its protective effect against anthracyclines has been the subject of animal experiments showing promising results [16, 17]. However, no randomized clinical trial has been conducted on human subjects yet.

The objective of the present study is to investigate the cardioprotective role of DFO in newly diagnosed pediatric cancer patients treated with DXO using echocardiography and serum markers such as N-terminal pro-brain natriuretic peptide (NT-proBNP) and cardiac troponin I (cTnI). They have been reported to be useful markers in the detection of DOX-induced cardiac tissue damage [18, 19].

## Methods

This single-center, parallel-group, open-label, randomized clinical trial was conducted at Amir Oncology Hospital, affiliated with Shiraz University of Medical Sciences ……. This hospital is the largest referral tertiary-care center for treating oncology patients in the South of  Iran….. Patients were consecutively selected among newly diagnosed, treatment-naive pediatric cancer patients with the age range of 2–18 years. They were treated with DOX as part of their chemotherapy protocol. Patients having congenital heart disease, established heart failure, confirmed renal diseases, previous mediastinal radiotherapy, or pretreatment with any kind of chemotherapy were not eligible to enter the study. The benefits and harms of the intervention were clearly explained to the lawful guardians of participants, and informed written consent was obtained from volunteers. The study protocol was approved by the Ethics Committee of Shiraz University of Medical Sciences….. with the code number IR.SUMS.MED.REC.1394.83………. The local university supported us financially with Grant No. 93-01-01-8499. The study was also registered in the Iranian…. Registry of Clinical Trials with the registration code IRCT2016080615666N5. The study adheres to the CONSORT guidelines for reporting clinical trials, and a completed CONSORT checklist was completed and attached (Additional file 1).

Based on a previous study by Elvira et al. [20], the incidence of clinical and subclinical cardiac toxicity of DOX is 50%, which is reduced to 20% by dexrazoxane. Considering α=0.05, Power=70%, P1=55%, and P2=20%, the sample size was calculated to be at least 60.

**Fig. 1:**
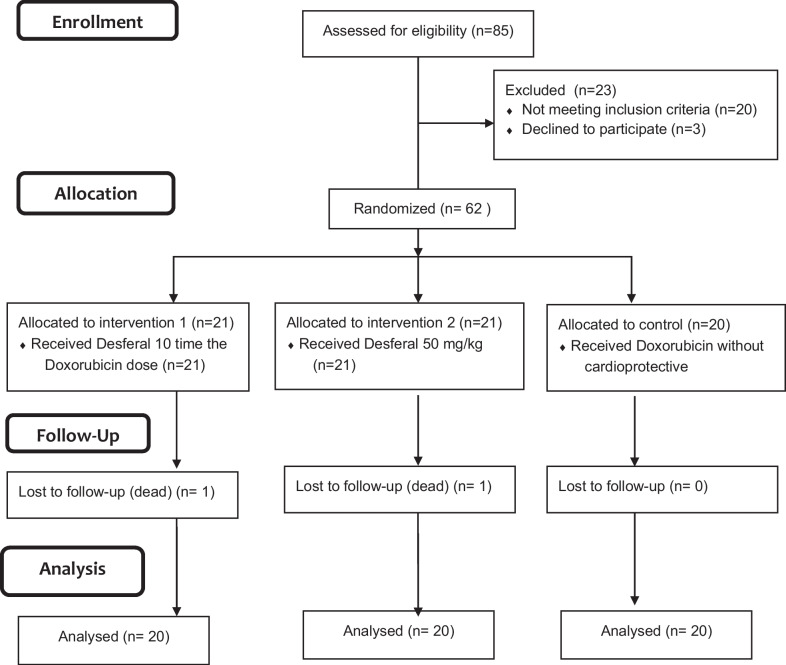
The study flow diagram

During 2016–2017 when the study was going on, every newly diagnosed patient who was admitted to the hospital and met the inclusion and exclusion criteria was interviewed. Those who took consent to take part in the trial were included. Sixty-two patients were recruited and were randomly allocated to 3 groups using a computer-generated block randomization sequence, which was done by a third party who was blind to the study protocol (Fig. 1). Neither the patients nor the investigators were aware of randomization except the cardiologist who performed echocardiography. Group 1 (n = 21) consisted of patients who received DOX without any cardioprotective agents. Patients allocated to group 2 (n = 20) were pretreated with DFO (Desferal®, Novartis, Switzerland) 10 times the DOX dose, similar to the dose of dexrazoxane. Patients in group 3 (n = 21) were pretreated with DFO 50 mg/kg, which is the standard iron-chelation dosage for thalassemia patients. Intravenous infusion of DFO was started 2 h before starting chemotherapy, continued during DOX infusion (at least 4 h), and for another 2 h after termination of the infusion, making up a total of 8 h. This regimen was repeated each time for the patients referred to receive DOX as part of their chemotherapy courses. After the last course of chemotherapy, M-mode, two-dimensional Doppler, and tissue Doppler echocardiography were performed on all participants by an experienced pediatric cardiologist. In the M-mode echocardiogram, left ventricular internal diameter (LVID), left ventricular posterior wall (LVPW), and interventricular septum (IVS) in systole and diastole were measured. In Doppler echocardiography, early diastolic velocity (E) and late diastolic velocity (A) of mitral and tricuspid valves were measured. In pulse tissue Doppler, systolic velocity, early diastolic velocity (Ea), and late diastolic velocity (Aa) were measured at the lateral mitral annulus, lateral tricuspid annulus, and septum. An HS-70 Samsung echocardiography machine (South Korea, Samsung) with a 2-to-4-MHZ probe was used for doing echocardiography. Serum concentrations of NT-proBNP (enzyme-linked immunosorbent assay, bioassay technology laboratory kit, China) and cTnI (enzyme-linked fluorescent assay, Vidas kit, France) as markers of cardiac tissue injury and carditis were measured after completion of the last course of DOX infusion. The primary outcome was the comparison of the echocardiographic parameters and serologic markers of cardiac tissue damage between the intervention and control groups.

Data were analyzed using Windows SPSS Software, version 21. The Shapiro–Wilk test was used to examine the normality of continuous variables. Qualitative data were analyzed by Chi-square test. The comparison of quantitative data between two groups was done by Student's t-test and among three or more groups by ANOVA and post hoc tests. In the case of non-normal data, appropriate nonparametric tests were applied. P-value < 0.05 was considered statistically significant.

## Results

### Population characteristics

During the study, one patient in the control group died of cancer after the second course of chemotherapy. Another patient in group 3 withdrew his consent and was excluded from the study. Therefore, the trial was completed with 60 patients (n = 20 in each group) (Fig. 1). The mean age of participants was 7.38 ± 4.38 years, including 41 males and 19 females. There was no significant difference between the three groups concerning baseline parameters including age, body surface area, treatment duration, and DOX dosage. The cumulative dose of DOX ranged from 25 to 360 mg/m^2^ based on the treatment protocol. Table 1 summarizes the characteristics of the patients.

**Table 1:** Demographic and disease characteristics of the study patients

Parameters	Group 1 (n = 20)	Group 2 (n = 20)	Group 3 (n = 20)	P_1,2_	P_1,3_	P_2,3_	ANOVA
Age (year)	6.85 ± 4.92	8.17 ± 4.76	8.69 ± 4.89	0.40	0.27	0.75	0.50
Body surface area (m^2^)	0.94 ± 0.41	0.94 ± 0.34	1.05 ± 0.33	0.96	0.40	0.38	0.63
Treatment duration (month)	8.70 ± 2.20 (6–15)	7.49 ± 1.43 (6–11)	7.81 ± 1.42 (6–11)	0.22	0.17	0.79	0.25
Doxorubicin dosage (mg)	189.20 ± 127.5	232.50 ± 99.16	204 ± 108.6	0.25	0.70	0.44	0.49

Group 1: control group; group 2: intervention with deferoxamine 10-times the doxorubicin dose; group 3: intervention with deferoxamine 50 mg/kg

### Clinical status

During the study, no patient showed clinical or dose-limiting cardiotoxicity due to DOX treatment. In addition, we did not observe any clinical adverse effects of DFO administration.

### Serum markers

The values of cTnI were < 0.01 ng/ml in all our patients. Serum concentrations of NT-proBNP were 545 ± 1018, 97.5 ± 8.5, and 167.14 ± 841 pg/ml in groups 1, 2, and 3, respectively. According to the T-test, there was a significant difference between groups 1 and 3 (P 1,2 = 0.76, P 1,3 = 0.036, P 2,3 = 0.83).

### Echocardiography

M-mode echocardiography revealed that ejection fraction differed significantly between groups 1 and 2 (Table 2). The parameters of Doppler echocardiography were not significantly different among the 3 groups (Table 3). In pulse tissue Doppler echocardiography, significantly lower values of tissue Doppler late diastolic velocity at the lateral annulus of the tricuspid valve (AaT) were noticed in group 3 in comparison with controls (Table 4).

**Table 2:** M-mode echocardiography parameters in the study patients

Parameters	Mean ± SD			P-value			
	Group 1 (n = 20)	Group 2 (n = 20)	Group 3 (n = 20)	Groups1 & 2	Groups2 & 3	Groups1 & 3	ANOVA
IVSD	0.72 ± 0.29	0.69 ± 0.18	0.71 ± 0.20	0.695	0.792	0.853	0.916
LVIDD	3.97 ± 0.83	3.84 ± 0.43	29.17 ± 97.61	0.618	0.359	0.326	0.401
LVPWD	0.68 ± 0.21	0.72 ± 0.16	0.69 ± 0.18	0.600	0.606	0.956	0.843
EDV	73.14 ± 37.80	63.80 ± 19.01	69.20 ± 20.35	0.428	0.477	0.725	0.670
IVSS	1.06 ± 0.47	0.89 ± 0.22	1.03 ± 0.30	0.236	0.184	0.859	0.424
LVIDS	2.36 ± 0.56	2.35 ± 0.34	2.20 ± 0.48	0.991	0.363	0.437	0.620
LVPWS	0.80 ± 0.15	0.80 ± 0.25	0.83 ± 0.18	0.990	0.761	0.672	0.920
ESV	21.16 ± 11.50	19.99 ± 6.79	17.70 ± 9.51	0.741	0.477	0.376	0.606
SV	52.17 ± 28.16	43.78 ± 16.81	52.17 ± 15.26	0.357	0.178	1.000	0.493
EF	71.50 ± 8.12	68.00 ± 10.27	75.52 ± 8.59	0.323	0.045	0.198	0.098
FS	27.57 ± 17.91	15.66 ± 49.80	11.79 ± 45.36	0.722	0.481	0.181	0.233

Group 1: control group; group 2: intervention with deferoxamine 10-times the doxorubicin dose; group 3: intervention with deferoxamine 50 mg/kg

*IVSD* Interventricular septal thickness in diastole, *LVIDD* Left ventricular internal dimension at end-diastole, *LVPWD* Left ventricular posterior wall in diastole, *EDV* End d diastolic volume, *LVPWS* Left ventricular posterior wall in systole, *IVSS* Interventricular septal thickness in systole, *LVIDS* Left ventricular internal dimension at end systole, *ESV* End-systole volume, *SV* Stroke volume, *EF* Ejection fraction, *FS* Fraction shorten

**Table 3:** Doppler echocardiography parameters in the study patients

Parameters	Mean ± SD			P-value			
	Group 1 (n = 20)	Group 2 (n = 20)	Group 3 (n = 20)	Groups 1 & 2	Groups 2 & 3	Groups 1 & 3	ANOVA
EM	101.0 ± 29.34	108.3 ± 14.07	100.2 ± 10.51	0.433	0.105	0.926	0.543
AM	74.37 ± 24.04	83.5 ± 24.2	71.84 ± 20.73	0.334	0.196	0.764	0.410
ET	79.94 ± 17.49	75.49 ± 26.75	70.75 ± 10.14	0.607	0.544	0.098	0.430
AT	58.01 ± 12.93	49.07 ± 19.73	57.81 ± 16.64	0.168	0.232	0.971	0.302
PAT	121.4 ± 17.99	117.8 ± 38.88	120.2 ± 20.56	0.758	0.847	0.873	0.942
E/AM	1.41 ± 0.51	1.39 ± 0.42	1.50 ± 0.45	0.899	0.535	0.639	0.818

Group 1: control group; group 2: intervention with deferoxamine 10-times the doxorubicin dose; group 3: intervention with deferoxamine 50 mg/kg

*EM* early diastolic velocity of mitral valve, *AM* late diastolic velocity of mitral valve, *ET* early diastolic velocity of tricuspid valve, *AT* late diastolic velocity of tricuspid valve, *PAT* Pulmonary acceleration time, *E/AM* early-to-late diastolic velocity of mitral valve

**Table 4:** Tissue Doppler echocardiography parameters in the study patients

Parameters	Mean ± SD			P-value			ANOVA
	Group 1 (n = 20)	Group 2 (n = 20)	Group 3 (n = 20)	Groups1 & 2	Groups2 & 3	Groups1 & 3	
SM	11.73 ± 6.22	8.64 ± 1.48	9.22 ± 3.4	0.082	0.592	0.195	0.151
EaM	14.54 ± 4.45	16.35 ± 4.04	10.42 ± 12.61	0.286	0.116	0.265	0.173
AaM	7.42 ± 7.41	9.10 ± 4.33	7.02 ± 6.71	0.500	0.365	0.884	0.685
SS	10.30 ± 3.37	8.93 ± 1.74	8.71 ± 2.1	0.217	0.772	0.144	0.214
EaS	11.45 ± 2.97	12.39 ± 7.52	10.07 ± 7.67	0.691	0.445	0.496	0.637
AaS	95.22 ± 307.6	8.09 ± 5.02	7.92 ± 5.18	0.338	0.933	0.326	0.362
ST	16.42 ± 4.73	14.02 ± 2.7	17.98 ± 18.7	0.134	0.475	0.756	0.678
EaT	15.00 ± 4.37	17.84 ± 4.58	15.66 ± 9.17	0.113	0.463	0.804	0.512
AaT	16.45 ± 4.73	13.85 ± 7.00	10.86 ± 7.57	0.285	0.316	0.035	0.107
EM/EaM	8.14 ± 4.78	7.13 ± 2.49	5.35 ± 5.38	0.514	0.304	0.151	0.254

Group 1: control group; group 2: intervention with deferoxamine 10-times the doxorubicin dose; group 3: intervention with deferoxamine 50 mg/kg

*SM* tissue Doppler systolic velocity of mitral valve, *EaM* early diastolic velocity at lateral annulus of mitral valve, *AaM* late diastolic velocity at lateral annulus of mitral valve, *SS* systolic velocity of septum, *EaS* early diastolic velocity of septum, *AaS* late diastolic velocity of septum, *ST* systolic velocity of tricuspid valve, *EaT* early diastolic velocity at lateral annulus of tricuspid valve, *AaT* late diastolic velocity at lateral annulus of tricuspid valve, *EM/EaM* early diastolic velocity of mitral to early diastolic velocity at lateral annulus of mitral valve

By using Pearson analysis, only tissue Doppler systolic velocity of the septum (SS) showed a marginally significant negative correlation with the DOX dose and duration of treatment (p-value = 0.050, r = − 0.308). None of the other analyzed factors proved to be significant (Table 5).

**Table 5:** Correlation of duration of treatment and doxorubicin dose with echocardiography parameters

Parameters	Doxorubicin dosage		Duration of treatment	
	Pearson correlation	P-value	Pearson correlation	P-value
IVSD	− 0.032	0.838	− 0.112	0.474
LVIDD	0.055	0.725	− 0.003	0.986
LVPWD	0.161	0.303	0.088	0.576
EDV	0.124	0.426	0.014	0.931
IVSs	0.017	0.916	0.036	0.818
LVIDs	0.186	0.231	0.011	0.942
LVPWS	− 0.087	0.578	− 0.109	0.485
ESV	0.177	0.256	0.029	0.851
SV	0.064	0.682	− 0.010	0.949
EF	− 0.190	0.222	− 0.081	0.605
FS	0.250	0.389	0.360	0.206
EM	0.081	0.613	0.002	0.989
AM	0.024	0.882	− 0.139	0.385
ET	− 0.041	0.801	0.026	0.872
AT	0.116	0.469	0.162	0.310
PAT	− 0.191	0.244	− 0.042	0.800
SM	− 0.178	0.264	− 0.035	0.830
EAM	− 0.111	0.490	− 0.120	0.454
AaM	− 0.252	0.121	− 0.261	0.109
SS	− 0.308	0.050	− 0.0174	0.276
EaS	− 0.160	0.319	− 0.164	0.306
Aas	0.229	0.160	0.214	0.192
ST	0.129	0.423	0.196	0.220
EaT	0.154	0.338	0.144	0.369
AaT	− 0.177	0.228	− 0.078	0.640
E/AM	0.066	0.681	0.127	0.427
EM/Eam	− 0.046	0.776	− 0.066	0.680

*AaM *late diastolic velocity at lateral annulus of mitral valve, *AaS* late diastolic velocity of septum, *AaT* late diastolic velocity at lateral annulus of tricuspid valve, *AM* late diastolic velocity of mitral valve, *AT* late diastolic velocity of tricuspid valve, *EaM* early diastolic velocity at lateral annulus of mitral valve, *EaS* early diastolic velocity of septum, *EaT* early diastolic velocity at lateral annulus of tricuspid valve, *EDV* End diastolic volume, *EM* early diastolic velocity of mitral valve, *ET* early diastolic velocity of tricuspid valve, *E/AM* early to late diastolic velocity of mitral valve, *EF* Ejection fraction, *EM/EaM* early diastolic velocity of mitral to early diastolic velocity at lateral annulus of mitral valve, *ESV* End-systole volume, *FS* Fraction shortening, *IVSD* Interventricular septal thickness in diastole, *IVSS* Interventricular septal thickness in systole, *LVIDD* Left ventricular internal dimension at end-diastole, *LVIDS* Left ventricular internal dimension at end systole, *LVPWD* Left ventricular posterior wall in diastole, *LVPWS* Left ventricular posterior wall in systole, *PAT* Pulmonary acceleration time, *SM* tissue Doppler systolic velocity of mitral valve, *SS* systolic velocity of septum, *ST* systolic velocity of tricuspid valve, *SV* Stroke volume

## Discussion

This is the first randomized clinical trial in childhood cancer investigating the role of DFO in preventing DOX-induced cardiotoxicity. We showed that patients treated with a higher dose of DFO (50 mg/kg) had significantly lower levels of NT-proBNP compared to the control group, which might be indicative of the protective role of DFO at the molecular level. The rise in NT-proBNP level has been reported as one of the best markers for the initial stages of cardiac damage and can raise suspicion of the development of cardiotoxicity before it can be detected by echocardiographic assessment [21]. Thus, NT-proBNP is considered a valuable marker in the long-term follow-up of subclinical DOX-induced cardiotoxicity [18]. It should be noted that as all our patients had cTnl serum levels below the normal cutoff point, no analysis could be done. Although nobody showed clinical or serologic evidence of carditis, higher values of NT-proBNP in non-DFO-treated patients need further consideration. Furthermore, echocardiography findings indicated that DFO exerts some degree of protection against cardiac damage regarding higher ejection fraction (EF) in patients treated with high-dose DFO (50 mg/kg), though all EFs were within the normal range. One simple explanation is that none of our patients developed overt cardiotoxicity so their EFs remained in the normal range.

As mentioned earlier, the cardiotoxicity of DOX is mainly due to the iron-mediated formation of ROS and the promotion of myocardial oxidative stress. Moreover, oxidation–reduction chain of molecular interactions, altered mitochondrial protein expression, increased expression of atrial natriuretic peptide (ANP), and brain natriuretic peptide (BNP) genes leading to cardiac hypertrophy, degradation of myofilaments and cytoskeletal proteins may cause cardiomyopathy [5, 6, 22–24].

In addition, despite the tremendous efforts in detecting other protective agents, dexrazoxane remains the standard treatment in the prevention of ROS formation. Accordingly, a study by Popelova et al. sought to evaluate the efficacy of deferiprone, a traditional oral iron-chelator agent, in reducing chronic anthracycline cardiotoxicity in a rabbit model [25]. The authors reported that this medication did not show significant protection against cardiac dysfunction, morphological cardiac damage as well an increase in plasma cTnI [12].

Based on the well-established mechanism of iron-induced oxidation, we hypothesized that DFO might efficiently prevent iron-mediated cardiac damage in DOX-treated patients. Our hypothesis was advocated by several previous non-human studies. Al-Harbi and his coworkers tried to assess biochemical and histopathological aspects of DFO protection against cardiac and hematologic toxicities of DOX in a rat model [26]. The induced myocardial damage was recovered by the administration of this iron-chelator agent. The authors concluded that the clinical use of DFO could be promising [26]. In another animal study by Al-Shabanah et al. on rats, it was reported that DOX potentiates apoptosis via upregulation of CDKN2A and p53, and suppression of Mdm2 gene expression, while the preventive effect of DFO against DOX-induced cardiotoxicity is regulated via the TGF-β1/Smad pathway [16]. The protective effect of DFO against oxidative damage induced by doxorubicin in rat hearts, liver, and kidneys was studied by Saad et al. [27]. Pretreatment of rats with DFO 10 times the dose of DOX considerably reduced biochemical variables and tissue damage in histopathological evaluation. Authors concluded that DFO protects against acute DOX-induced cardiotoxicity in a dose-dependent manner [27].

The substantial difference in efficacy of dexrazoxane compared to other iron chelators may lie in other protective mechanisms. Jirkovsky et al. [28] suggested that DFO-induced depletion of TOP2b may be important for cardioprotection rather than the prevention of iron-related oxidative stress[28]. Another study emphasizing the distinct effects of these two agents was conducted by Cermanova et al. on the development of acute toxin-induced liver injury in rats [29]. It was stated that despite the reduction of liver iron content by both drugs, only DFO showed a protective effect against liver injury. Dexrazoxane surprisingly worsened the GSH/GSSG ratio, which is an indicator of oxidative stress in the tissues [29]. A comparison of ICRF-187 and DFO in their protective role against chronic cardiac toxicity due to DOX in hypertensive rats showed that the latter agent provided a better degree of protection [17].

Our study had some limitations that might have influenced the result of our investigation. First, the short-term follow-up of our patients allowed us to conclude the acute and subacute toxicities of DOX while we couldn’t judge the late DOX toxicities and the preventive role of DFO on them. Second, as DOX cardiotoxicity occurs in a proportion of patients, the small number of participants could affect the results of our study. In addition, the study population was not homogeneous in terms of the chemotherapy protocol, and other cardiotoxic agents in the treatment regimen beyond DOX might have had an impact on the final result, though their effects were negligible. Finally, we did not measure serum biomarkers of cardiac toxicity before starting the study due to financial limitations and just measured them at the end of the study. Moreover, baseline echocardiography was performed just by conventional M-mode echo, and tissue Doppler echo was conducted after the last dose of DOX.

Despite these limitations, our study is unique, given that it is the first clinical trial conducted on true patients assessing the efficacy of DFO with different doses in preventing cardiac toxicity of DOX. Though we could not prove the beneficial role of DFO in the short term, it seems promising that larger multicenter studies with longer follow-ups might show its efficacy for preventing chronic DOX toxicity. Besides, we suggest that the efficacy of DFO and dexrazoxane be compared in future trials.

## Conclusions

DFO may have a minor role in preventing the acute cardiotoxicity of DOX. Its efficacy in preventing delayed toxicities warrants further studies with larger sample sizes and longer follow-ups. In addition, it is better to use imaging modalities such as functional cardiac magnetic resonance imaging (MRI) and tissue Doppler as well as speckle echocardiography to elucidate more clearly if it has any beneficial role in cardiac protection against anthracyclines. It can be assumed that the role of iron in anthracycline cardiotoxicity is complex and that other sophisticated mechanisms might be responsible for the protective role of each particular iron chelator.

## Supplementary Information

**Additional file ﻿1**. The CONSORT Checklist.

## Data Availability

The data are available upon request of the journal.

## Abbreviations

ANP: Atrial natriuretic peptide
BNP: Brain natriuretic peptide
cTnI: Cardiac troponin I
DFO: Deferoxamine
DOX: Doxorubicin
EF: Ejection fraction
IVS: Interventricular septum
LVID: Left ventricular internal diameter
LVPW: Left ventricular posterior wall
MRI: Magnetic resonance imaging
NT-pro BNP: N-terminal pro-brain natriuretic peptide
ROS: Reactive oxygen species
SI: Serum iron
SS: Systolic velocity of the septum
